# Deep sedation vs. general anesthesia for transcatheter tricuspid valve repair

**DOI:** 10.3389/fcvm.2022.976822

**Published:** 2022-08-31

**Authors:** Jean Marc Haurand, Refik Kavsur, Laurin Ochs, Tetsu Tanaka, Christos Iliadis, Atsushi Sugiura, Malte Kelm, Georg Nickenig, Stephan Baldus, Ralf Westenfeld, Marc Ulrich Becher, Roman Pfister, Patrick Horn

**Affiliations:** ^1^Division of Cardiology, Pulmonology and Vascular Medicine, Medical Faculty, University Düsseldorf, Düsseldorf, Germany; ^2^Department of Cardiology, Angiology, Pneumology and Medical Intensive Care, University Hospital Bonn, Bonn, Germany; ^3^Department of Cardiology, Angiology, Pneumology and Medical Intensive Care, Heart Center of the University of Cologne, Cologne, Germany

**Keywords:** tricuspid valve regurgitation, TriClip, conscious sedation, general anesthesia, deep sedation

## Abstract

**Background:**

Transcatheter tricuspid valve repair (TTVr) is routinely performed under general anesthesia (GA). This study aimed to investigate whether TTVr procedures can be performed effectively and safely without GA but using deep sedation (DS).

**Methods:**

We performed a retrospective analysis of 104 patients from three centers who underwent TTVr between 2020 and 2021. The primary performance endpoints were technical success and severity of TR assessed at the time of discharge. The safety outcome was a composite of in-hospital complications, including occurrence of death, conversion to surgery, major adverse cardiac and cerebrovascular events, major vascular complications, or occurrence of pneumonia.

**Results:**

Sixty-four procedures were performed in GA and 40 procedures were performed in DS. The groups did not differ in age, EuroScore II, TR severity, ventricular function, or hemodynamic parameters. Technical success was achieved in 92.5% of the patients in the DS group and in 93.6% of the patients in the GA group (*p* = 0.805). In none of the patients intraprocedural conversion from DS to GA was required. There was no difference in total duration of the procedure, and number of devices implanted. The degree of TR was ≤2+ in 77.5% of the patients in the DS group and in 74.2% of the patients in the GA group (*p* = 0.705). The composite safety endpoint did not differ between the groups (2.5 vs. 6.3%, *p* = 0.384). The total duration of hospital stay was shorter in patients who underwent TTVr in DS compared to those who underwent TTVr in GA (6 [5, 9] days vs. 8 [6, 11] days; *p* = 0.011).

**Conclusion:**

Performing TTVr in DS was effective with similar procedural results, and was safe with similar low complication rates compared to GA.

## Introduction

Transcatheter tricuspid valve repair (TTVr) is a promising therapy of severe tricuspid regurgitation (TR) (1, 2). The TTVr procedure is routinely performed under general anesthesia (GA), which provides a controlled setting for this complex and transesophageal echocardiography (TEE)-guided procedure. However, the typical candidate for TTVr is of advanced age and is characterized by various comorbidities, turning him/her into a patient at risk under GA as hypotension with the need for vasopressor agents or prolonged need for invasive ventilation (3).

For transcatheter edge-to-edge repair of the mitral valve, which is another complex TEE-guided procedure, the feasibility and safety of deep sedation (DS) without the need for endotracheal intubation or mechanical ventilation have been demonstrated (4–6). However, imaging during TTVr is much more challenging and requires switching back and forth from transgastric to esophageal views (7). These changes in the intubation height of the TEE probe could be more strenuous for patients, and patient movements could hamper the procedure. The feasibility of performing TTVr using DS was demonstrated as a case report (8). This study aimed to compare for the first time the performance of TTVr procedures in GA and DS in terms of feasibility and safety and in multiple centers with a high level of experience.

## Methods

The analysis was a multicenter observational study using the data from the Heart Failure Network Rhineland registry (University Hospitals Bonn, Cologne, and Duesseldorf). In this study, 104 patients from three centers with significant TR who were treated with the TriClip^®^ device (Abbott, Vascular GmbH) between September 2020 and September 2021 were included. Patients were grouped according to the performance of TTVr in GA or DS. The type of anesthesia was determined by the center in which the TTVr procedure was performed. GA was used as the standard of care at the University Hospitals Cologne and Bonn, and DS was used as the standard of care at the University Hospital Düsseldorf. The study was approved by the institutional review board of the individual centers and was conducted in accordance with the Declaration of Helsinki. All patients provided written informed consent to participate in the registry. Severe TR was defined according to the European Society of Cardiology’s Guidelines for the management of valvular heart disease (9).

In the GA group, anesthetic induction was performed with a bolus dose of etomidate or propofol, fentanyl, and non-depolarizing muscle relaxants to facilitate oral tracheal intubation using an appropriately sized endotracheal tube. Anesthesia was maintained using sevoflurane or desflurane. The patients were prepared with a radial arterial catheter for hemodynamic measurements and central venous catheterization to supply the medication (saline, catecholamines, propofol, sufentanil, and remifentanil). Echocardiographic guidance of the procedure was performed by a cardiologist. The patients were extubated on the table in the catheterization laboratory or in the recovery room. After the procedure, all patients were transferred to the intermediate care unit (IMCU) or intensive care unit (ICU).

In the DS group, all patients received local anesthesia with 10 mL of 0.2% lidocaine administered subcutaneously at the femoral access site. Hemodynamic monitoring and measurements were performed using a radial arterial catheter. All patients received 2–3 mg of midazolam 30 min prior to the procedure. In addition to hemodynamic and respiratory monitoring, the level of sedation was assessed and monitored using the Richmond Agitation-Sedation Scale (RASS) with the goal of a score of minus 3 to ensure an adequate level of sedation for a safe procedure. Sedation was initiated by administering a propofol bolus adjusted to the patient’s actual body weight. Half of the calculated bolus was initially administered, followed by a partial dose of the remaining amount within 5 min while monitoring the hemodynamic and respiratory responses as well as the depth of sedation according to the RASS score. If the desired sedation level was not achieved after the initial bolus, propofol boluses (0.25 mg/kg) were administered further until the sedation level was reached. When the desired sedation level was achieved, sedation resumed by continuous administration of propofol at 1.5 mg/kg/h according to the RASS score and hemodynamic and respiratory monitoring. Patients were monitored by a cardiologist with more than 12 months of training in intensive care medicine. The cardiologist was also responsible for the echocardiographic guidance of the procedure. After the procedure, all patients were monitored in the recovery room of the catheter lab until they had fully woken up and were brought to the general ward or IMCU.

The TriClip^®^ device and procedure have been previously described (10). The clip delivery system was introduced into the guide catheter, and the TriClip^®^ device was advanced into the vena cava. The clip delivery system was guided to the tricuspid valve, where the leaflets were grasped, and the clip was deployed.

The endpoints of this study were defined according to the Mitral Valve Academic Research Consortium (MVARC) unless otherwise indicated (11). Clinical endpoints and echocardiographic results were assessed by the local investigators. The safety outcome was a composite of in-hospital complications, including occurrence of death, periprocedural conversion from DS to GA, conversion to surgery, myocardial infarction, cerebrovascular events, major vascular complications, or occurrence of pneumonia. Minor vascular complications were defined as minor vascular and bleeding complications according to the MVARC (11). The primary performance endpoints were technical success and severity of TR assessed at the time of discharge. Procedure time was defined as the time from vascular puncture to closure of femoral access. All data collection was performed retrospectively with the approval of the Institutional Review Board of the respective academic center, and the central analysis was based on anonymized data.

### Statistics

Categorical variables are reported as absolute values and percentages, whereas continuous data are expressed as median with interquartile range. The D’Agostino and Pearson omnibus normality test was used to assess the non-parametric characteristics of the parameters. Patient characteristics were compared using the Mann–Whitney *U* test (continuous data), and using the two-tailed Fisher’s exact and Pearson’s chi-squared test (categorical data). Statistical significance was set at *p* < 0.05. We used pairwise deletion, respectively, listwise deletion methods to eliminate missing data. Statistical analyses were performed using SPSS^®^ Statistics 28 (IBM^®^) and Prism^®^ (GraphPad^®^).

## Results

**FIGURE 1:**
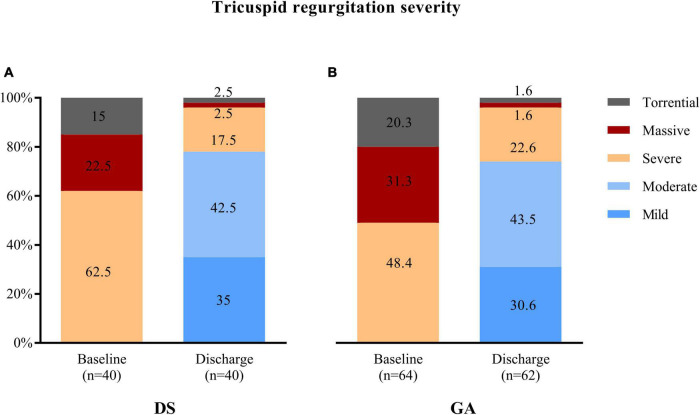
Tricuspid regurgitation (TR) severity was similarly reduced at discharge after transcatheter tricuspid valve repair using **(A)** deep sedation (DS) or **(B)** general anaesthesia (GA).

TTVr was performed in 40 patients with DS at one center and 64 patients with GA at two centers. The two groups had comparable baseline characteristics and comorbidities (Table 1). The median age was 81 (77, 83) years in the DS group and 80 (76, 84) years in the GA group (*p* = 0.917). The EuroScore II was similarly high in both groups [5.7 (3.1, 9.9)% vs. 5.7 (3.6, 10.2)%, *p* = 0.859]. Only the presence of diabetes mellitus was higher in the DS group than in the GA group (30 vs. 12.5%, *p* = 0.028). Additionally, the groups did not differ in echocardiography-derived ventricular and valvular parameters (Table 2). All patients had severe-to-torrential TR at baseline (Figure 1). Patients in both groups also had similar left ventricular and right ventricular function measurements (left ventricular ejection fraction: 56 [48, 64]% in the DS group vs. 55 [51, 58]% in the GA group, *p* = 0.115; tricuspid anterior plane systolic excursion: 17 [13, 18] mm in the DS group vs. 17 [13, 20] mm in the GA group, *p* = 0.148). Furthermore, 87% of the patients in the DS group and 76.5% of the patients in the GA group had New York Heart Association (NYHA) functional class III or IV (*p* = 0.168).

**TABLE 1:** Characteristics of TTVr patients grouped according to the anesthesia (GA or DS) used. Categorical variables are reported as absolute values and percentages, whereas continuous data are expressed as median with interquartile range.

	DS (*n* = 40)	GA (*n* = 64)	*p*-value
**Baseline characteristics**			
Age (years)	81 (77, 83)	80 (76, 84)	0.917
Body mass index (kg/m^2^)	24.7 (21.8, 29.2)	24.7 (22.0, 26.8)	0.707
Female, n (%)			0.132
EuroSCORE II (%)	26 (65.0)	32 (50.0)	0.859
	5.7 (3.1, 9.9)	5.7 (3.6, 10.2)	
NYHA functional class, n (%)			0.359
II	5 (12.5)	15 (23.4)	
III	31 (77.5)	42 (65.6)	
IV	4 (10.0)	7 (10.9)	
**Comorbidities, n (%)**			
Arterial hypertension	30 (75)	38 (59.4)	0.103
Diabetes mellitus	12 (30)	8 (12.5)	0.028*
Previous myocardial infarction	3 (7.5)	13 (20.3)	0.078
Previous cardiac bypass surgery	5 (12.5)	8 (12.5)	0.999
Previous mitral valve surgery	4 (10.0)	8 (12.5)	0.542
Pacemaker/ICD/CRT	5 (12.5)	17 (26.6)	0.088
Atrial fibrillation	35 (87.5)	57 (89.0)	0.808
Chronic lung disease	8 (20.0)	11 (17.2)	0.718
Previous Stroke	4 (10.0)	6 (9.4)	0.999
Estimated GFR (ml/min)	46 (32, 68)	42 (30, 64)	0.229
NT-proBNP (*1,000 pg/ml)	2.1 (1.2, 2.8)	2.0 (1.3, 4.6)	0.174
ALT (IU/l)	21 (14, 25)	20 (14, 26)	0.699
Diuretic medication, n (%)	38 (95.0)	59 (92.2)	0.578
Beta blocker medication, n (%)	33 (82.5)	54 (84.4)	0.801

* indicates *p* ≤ 0.05. TTVr, Transcatheter tricuspid valve repair; DS, Deep sedation; GA, General anesthesia; NYHA, New York Heart Association; ICD, internal cardiac defibrillator; CRT, cardiac resynchronization therapy; GFR, glomerular filtration rate; NT-proBNP, brain natriuretic peptide; ALT, Alanine Aminotransferase; TAVR, transcatheter aortic valve replacement. The significant values are in bold.

**TABLE 2:** Baseline echocardiographic and hemodynamic parameters of TR patients grouped according to the device system used for TTVr.

	DS (*n* = 40)	GA (*n* = 64)	*p*-value
TR severity, n (%)			0.557
Moderate	2 (5.0)	1 (1.6)	
Severe	38 (95.0)	63 (98.4)	
Left ventricular ejection fraction (%)	56 (48, 64)	55 (51, 58)	0.115
TAPSE (mm)	17 (13, 18)	17 (13, 20)	0.148
Tricuspid valve annulus (mm)	41 (38, 45)	42 (40, 46)	0.415
TR vena contracta (mm)	8.9 (7.0, 12.4)	10.0 (7.8, 13.9)	0.184
TR EROA (mm^2^)	47 (30, 64)	50 (40, 57)	0.432
TR volume (ml)	45 (39, 70)	51 (40, 64)	0.537
TR max pressure gradient (mmHg)	34 (24, 47)	31 (25, 42)	0.651
Hemodynamic parameters			
PAP systolic (mmHg)	45 (35, 59)	44 (34, 55)	0.969
PAP mean (mmHg)	31 (22, 35)	29 (21, 36)	0.801
PAP diastolic (mmHg)	17 (10, 23)	17 (12, 24)	0.402

Categorical variables are reported as absolute values and percentages, whereas continuous data are expressed as median with interquartile range. TTVr, Transcatheter tricuspid valve repair; DS, Deep sedation; GA, General anesthesia; TR, Tricuspid regurgitation; EROA, Effective regurgitation orifice area; TAPSE, Tricuspid annular plane systolic excursion; PAP, Pulmonary artery systolic pressure.

Successful device implantation of at least one device and reduction of TR of at least one degree was achieved in 37 out of 40 patients (92.5%) in the DS group and in 60 out of 64 patients (93.6%) in the GA group (*p* = 0.805). The reasons for technical failure included the inability to grasp the leaflets, inability to verify adequate leaflet insertion due to low image quality, or inability to adequately reduce TR. The number of implanted devices was similar in both groups (*p* = 0.563) (Table 3). One device was implanted in 12 (30%) patients in the DS group and in 12 (19%) patients in the GA group. Two devices were implanted in 22 (55%) patients in the DS group and in 41 (64%) patients in the GA groups. Three devices were implanted in 3 (8%) patients in the DS group and in 7 (11%) patients in the GA group. The mean tricuspid pressure gradient measured post-procedural in the catheter laboratory was higher in the DS group compared to the GA group (2.0 [1.0, 2.0] mmHg vs. 1.3 [0.9, 1.9] mmHg; *p* = 0.028). The degree of TR assessed at the time of discharge was ≤2+ in 31 out of 40 (77.5%) patients in the DS group and in 46 out of 62 (74.2%) patients in the GA group (*p* = 0.705) (Figure 1). The duration of the procedure did not differ between the DS and GA groups (75 [64, 96] min vs. 80 [54, 100] min; *p* = 0.662) (Table 3).

**TABLE 3:** Procedural outcome categorical variables are reported as absolute values and percentages, whereas continuous data are expressed as median with interquartile range.

	DS (*n* = 40)	GA (*n* = 64)	*p*-value
Technical success, n (%)	37 (92.5)	60 (93.6)	0.805
Devices implanted, n (%)			0.563
0	3 (7.5)	4 (6.3)	
1	12 (30)	12 (18.7)	
2	22 (55)	41 (64.1)	
3	3 (7.5)	7 (10.9)	
Tricuspid transvalvular gradient at end of procedure (mmHg)	2.0 (1.0, 2.0)	1.3 (0.9, 1.9)	0.028*
Procedure time (min)	75 (64, 96)	80 (54, 100)	0.662
Postprocedural transfer to ICU/IMCU, n (%)	4 (10)	64 (100)	0.001*
Composite safety endpoint, n (%)	1 (2.5)	4 (6.25)	0.384
In-hospital mortality, n (%)	0 (0)	2 (3.1)	
Pneumonia, n (%)	1 (2.5)	2 (3.1)	
Major bleeding, n (%)	0 (0)	0 (0)	
Stroke, n (%)	0 (0)	0 (0)	
Myocardial infarction, n (%)	0 (0)	0 (0)	
Minor bleeding, n (%)	5 (12.5)	5 (7.8)	0.502
Acute kidney failure, n (%)	2 (5)	7 (10.9)	0.295
Length of ICU/IMCU stay (days)	0 (0, 0)	1 (1, 1)	0.001*
Length of hospital stay (days)	6 (5, 9)	8 (6, 11)	0.011*

* indicates *p* ≤ 0.05. DS, Deep sedation; GA, General anesthesia; IMCU, Intermediate Care Unit; ICU, Intensive Care Unit. The significant values are in bold.

After the procedure, all patients in the GA group were transferred to either the IMCU (42 patients) or ICU (22 patients) (Table 3). Patients who underwent TTVr in DS were observed after the procedure in the recovery room of the catheterization laboratory until the patients were fully awake. Afterward, 4 out of 40 (10%) patients were transferred to the IMCU (one patient due to early access site minor bleeding that could be resolved quickly by manual compression and three patients due to high risk of postprocedural delirium). The remaining 36 patients (90%) were transferred back to the general ward.

The composite safety endpoint of all-cause death, conversion to surgery, myocardial infarction, stroke, pneumonia, or major to life-threatening bleeding did not differ between the groups (1 [2.5%] patient in the DS group vs. 4 [6.3%] patients in the GA group, *p* = 0.384) (Table 3). In the DS group, conversion to GA was not necessary for any patient. In the DS group, one patient (2.5%) had a leaflet laceration obtained during clip deployment, whereas another patient (2.5%) had early single leaflet attachment (SLDA); both events could be fixed with a second device. In the GA group, SLDA occurred in two patients (3.1%) and could be fixed with additional devices. In the GA group, two patients (3.1%) died during intra-hospital stay that was not related to the procedure. The occurrence of pneumonia (one patient in the DS group [2.5%] and two patients in the GA group [3.1%]) and the occurrence of acute renal failure (one patient in the DS group [2.5%] and seven patients in the GA group [10.9%], *p* = 0.149) did not differ between the groups.

The total duration of hospital stay was shorter in patients who underwent TTVr in DS compared to those who underwent TTVr in GA (6 [5, 9] days vs. 8 [6, 11] days; *p* = 0.011) (Table 3).

## Discussion

Here, we demonstrated that in 104 patients from three centers, (i) performing TTVr in DS was effective with similar procedural results compared to performing TTVr in GA, and (ii) performing TTVr in DS was as safe as TTVr performed in GA.

Concerns regarding the performance of TTVr without GA include the possibly unprotected airway, the risk of aspiration, and undesired patient movements during the procedure. Patient movements could hamper the procedure or even risk vascular or cardiac lesions due to the stiff catheter or delivery system. In GA, the patient is still lying, without the risk of potential body movements, coughs, or gags while crossing the tricuspid valve with the device. In addition, device deployment may be facilitated by controlled respiration of the ventilator (12).

In this study, none of the patients in the DS group required conversion to GA, and no structural cardiac damage occurred due to potential body movements. The rate of pneumonia after the procedure did not differ between the DS and GA groups, indicating that aspiration was not an issue while performing TTVr using DS.

Successful clip implantation was achieved in 93% of the patients in the DS group, which was similar in the GA groups. The experience in performing TTVr was similar high in all centers. In our study, the numbers of procedures differ between the centers as we have included only TTVr procedures using the TriClip^®^ device to increase comparability. The average number of implanted clips, grade of TR reduction, and procedural time were comparable between TTVr performed in DS and TTVr performed in GA. The possibility of independent leaflet grasping might have facilitated maximum leaflet insertion and spans large coaptation gaps in severe TR without the need for ventilation maneuvers performed only under GA. These findings are consistent with those of previous studies demonstrating the safety and efficacy of DS for transcatheter mitral valve intervention (4–6). Therefore, TTVr performed in DS was as safe and effective as that performed in GA.

Performing DS is challenging because patients are typically characterized by advanced age, occasional right ventricular dysfunction, and pulmonary diseases that might hamper the assessment of the patient’s response to sedation (10, 12). In addition, imaging is more challenging in TTVr than in TEER of the mitral valve, as TTVr requires switches of transgastric and deep esophageal views that can stimulate patient movements when the sedation grade is not deep enough (7). It is notable, that the institution performing TTVr in DS in this study is a highly experienced center performing high numbers of procedures (including transcatheter vale replacement, left atrial appendage occlusion, ablation of atrial fibrillation) in cardio-analgosedation (5, 13). Though the level of experience in DS plays a prominent role, patient characteristics might predict a challenging DS procedure. It has been previously demonstrated that in patients with a higher body mass index (>31 kg/m^2^) TEER for the mitral valve might be difficult to perform using DS (drops of oxygen saturation, disruptive body movements) (14). In obese patients, the pharmacokinetics of drugs may be unpredictable and the volume of distribution is increased for lipid soluble agents such as propofol and fentanyl possibly resulting in the need for a higher dose of these agents to reach the target level of sedation (15). The challenge of DS in these patients is to maintain an adequate level of sedation, which assures an optimal procedural condition, and which avoids respiratory failure and hemodynamic compromise. Despite the safety of DS for TTVr which was demonstrated in this study, backup for immediate endotracheal intubation by an anesthesiologist or an intensive care physician is crucial.

In our study, most patients in the DS group were transferred to the normal ward after the procedure without a stay at the ICU or IMCU. Bypassing ICU/IMCU might have shorten the total length of hospital stay. However, the higher rate of ICU/IMCU stay in the GA centers might be based on different peri-procedural managements between the centers. GA was used as the standard of care at the University Hospitals Cologne and Bonn, and DS was used as the standard of care at the University Hospital Düsseldorf. Comparing TTVr using DS vs. GA at one center was not possible. Therefore, comparing post-procedural processes and management between the groups was limited.

Taken together, TTVR can be performed safely and effectively in DS, as in GA. It remains unclear which patient can benefit more from TTVr performed in DS instead of GA or vice versa. Each mode of anesthesia seems to have advantages and disadvantages. These could potentially affect the decision which mode of anesthesia should be used in which patient (Supplementary Table 1 and Supplementary Figure 1). However, this risk–benefit evaluation is based on theoretical considerations and subjective experience. In addition, our study could weaken some of these considerations (Supplementary Table 1). Further studies are required investigating which patients can benefit from a specific mode of anesthesia for TTVr.

This study had some further limitations. As this was not a randomized controlled study, the study inference may be biased among the three centers regarding patient selection and therapeutic management. In addition, since the number of patients included in the current analysis was small, the statistical power of detecting the difference might be limited. Furthermore, the grade of simplicity/complexity required to achieve sufficient DS in these patients was not assessed. Nonetheless, this is the first study investigating the safety and feasibility of DS during the TTVr procedure, showing similar periprocedural outcomes compared to GA. More studies are required to identify patients at risk for a difficult application of DS before this can be generally recommended for TTVr at institutions with less experience.

## Conclusion

We demonstrated that performing TTVr in DS was effective with similar procedural results, and was safe with similar low complication rates compared to performing TTVr in GA.

## Data availability statement

The raw data supporting the conclusions of this article will be made available by the authors, without undue reservation.

## Ethics statement

The study was approved by the Institutional Review Board of University Düsseldorf, University Bonn, and University Cologne and was conducted in accordance with the Declaration of Helsinki. The patients/participants provided their written informed consent to participate in this study.

## Author contributions

JH and PH wrote the original manuscript, performed formal analysis, and revised the manuscript. RK, LO, TT, CI, and AS were involved in data collection and contributed to manuscript review. MK, GN, SB, RW, MB, and RP were involved in supervision and manuscript review and editing. PH conceptualized the study and responsible for the overall content. All authors contributed to the article and approved the submitted version.

## Supplementary material

Supplementary Figure 1 Patient and center characteristics that might have an impact on the decision using deep sedation (DS) or general anesthesia (GA) for transcatheter tricuspid valve repair (TTVr).
