# Supplementary material for: Deep sedation vs. general anesthesia for transcatheter tricuspid valve repair
Source: Front Cardiovasc Med. 2022 Aug 31;9:976822. doi: 10.3389/fcvm.2022.976822 (PMC9471949; doi:10.3389/fcvm.2022.976822)
Supplement: Supplementary file 1 [file Table_1.DOCX]

**Supplemental Table 1.** Potential advantages and disadvantages of two modes of anesthesia (DS vs. GA) for performing TTVr.

|  | **DS** | **GA** |
| --- | --- | --- |
| **+** | Avoidance of GA and the associated potential risk of vasopressor agents use, prolonged need for invasive ventilation, and postprocedural cognitive dysfunction | Protected airway, no risk of aspiration  *(In our study: no conversion from DS to GA required, similar rates of pneumonia between the groups)* |
| **+** | Might allow bypassing ICU/ IMCU in the majority of cases  *(Immediate extubation on the operating table and post-procedural monitoring in a recovery room can also avoid the need for ICU admission after GA)* | Device deployment may be facilitated by controlled respiration of the ventilator  *(In our study: similar procedural results between the groups)* |
| **-** | Experience in DS procedures required to maintain an adequate level of sedation and to avoid respiratory failure and hemodynamic compromise | Risk of hypotension with the need for vasopressor agents or prolonged need for invasive ventilation |
| **-** | Potential risk of undesired patient movements during the procedure  *(In our study: similar procedural results and safety between the groups)* |  |

*TTVr = Transcatheter tricuspid valve repair; DS = Deep sedation; GA = General anesthesia; ICU = Intensive Care Unit; IMCU = Intermediate Care Unit*
