# Supplementary material for: Deep sedation vs. general anesthesia for transcatheter tricuspid valve repair
Source: Front Cardiovasc Med. 2022 Aug 31;9:976822. doi: 10.3389/fcvm.2022.976822 (PMC9471949; doi:10.3389/fcvm.2022.976822)
Supplement: Supplementary Figure 1 — Patient and center characteristics that might have an impact on the decision using deep sedation (DS) or general anesthesia (GA) for transcatheter tricuspid valve repair (TTVr). [file Image_1.PDF]

TTVr in DS	TTVR in GA
	Low experience of the center in performing procedures using DS
Severe LV dysfunction with risk of hypotension under GA	Obesity (Body mass index > 35 kg/m^2) with risk of challenging DS in regard to achieving an adequate level of sedation and avoiding of respiratory failure
Respiratory disease with risk of prolonged need for ventilation when using GA	Expected difficult airway management in case of conversion from DS to GA
Concomitant cognitive disorders or high risk of delirium / cognitive dysfunction after GA	Large gap of the tricuspid valve + interventionalist who like to use controlled respiration of the ventilator to facilitate device deployment
